# Biophysical Parameters Can Induce Epithelial-to-Mesenchymal Phenotypic and Genotypic Changes in HT-29 Cells: A Preliminary Study

**DOI:** 10.3390/ijms24043956

**Published:** 2023-02-16

**Authors:** Judith Pape, Auxtine Micalet, Wissal Alsheikh, Nadia Ezbakh, Rania-Iman Virjee, Rawiya Al Hosni, Emad Moeendarbary, Umber Cheema

**Affiliations:** 1 Centre for 3D Models of Health and Disease, Department of Targeted Intervention, Division of Surgery and Interventional Science, University College London, Charles Bell House, 43-45 Foley Street, London W1W 7TS, UK; 2 Department of Mechanical Engineering, University College London, Gower Street, London WC1E 6BT, UK

**Keywords:** tumour microenvironment, hypoxia, collagen, EMT, 3D model, colorectal cancer, tumour stiffness

## Abstract

Epithelial to mesenchymal transition (EMT) in cancer is the process described where cancer epithelial cells acquire mesenchymal properties which can lead to enhanced invasiveness. Three-dimensional cancer models often lack the relevant and biomimetic microenvironment parameters appropriate to the native tumour microenvironment thought to drive EMT. In this study, HT-29 epithelial colorectal cells were cultivated in different oxygen and collagen concentrations to investigate how these biophysical parameters influenced invasion patterns and EMT. Colorectal HT-29 cells were grown in physiological hypoxia (5% O_2_) and normoxia (21% O_2_) in 2D, 3D soft (60 Pa), and 3D stiff (4 kPa) collagen matrices. Physiological hypoxia was sufficient to trigger expression of markers of EMT in the HT-29 cells in 2D by day 7. This is in contrast to a control breast cancer cell line, MDA-MB-231, which expresses a mesenchymal phenotype regardless of the oxygen concentration. In 3D, HT-29 cells invaded more extensively in a stiff matrix environment with corresponding increases in the invasive genes *MMP2* and *RAE1*. This demonstrates that the physiological environment can directly impact HT-29 cells in terms of EMT marker expression and invasion, compared to an established cell line, MDA-MB-231, which has already undergone EMT. This study highlights the importance of the biophysical microenvironment to cancer epithelial cells and how these factors can direct cell behaviour. In particular, that stiffness of the 3D matrix drives greater invasion in HT-29 cells regardless of hypoxia. It is also pertinent that some cell lines (already having undergone EMT) are not as sensitive to the biophysical features of their microenvironment.

## 1. Introduction

The leading cause of mortality for cancer is metastasis [1]. The hallmark of cancer aggressiveness is the ability to invade and repopulate distant organs [2]. In this context, it is vital to understand how the physical tissue microenvironment directs cancer progression. With increased understanding of pathological processes and the tumour microenvironment (TME), disease models have evolved to include more biomimetic features that aim to closely mimic in vivo tissue [3]. Metastatic tumour progression is mediated by environmental conditions such as oxygen concentration (hypoxia) and matrix stiffness [4]. The use of 3D cancer models allows for a systematic understanding of how physical and biochemical features can change cancer cell progression and invasive phenotype. Using defined tissue models allows for the analysis of specific effects caused by extracellular matrix (ECM) components and spatial configurations of cancer cell growth and metastasis [5]. To mimic cancer cell behaviour in vivo, researchers are altering their culturing methods and models used. Cancer cells reside in a three-dimensional environment, within an extracellular matrix, among populations of healthy cells, stromal cells, immune cells, and vascular tissue [6]. Many techniques and materials have been used to bioengineer 3D cancer models to bridge the gap between conventional 2D cultures and the TME. The wide range of 3D cancer models used include but are not limited to scaffold-free cancer [7] spheroids, scaffold-based matrices, microfluidic systems, 3D bioprinting, and tumour organoids termed “tumouroids” [8]. These models allow for the modulation of various microenvironmental factors including ECM constituents, physical stiffness, and the addition of stromal cell populations [9]. There is a growing body of literature on the effect of physical stiffness on cancer invasion in 3D cancer models [10]. As cancer cells grow and nest among healthy tissue, they actively stimulate surrounding fibroblasts to modify the ECM into a stiffer, collagen-dominant matrix [11]. It is becoming increasingly critical to understand whether cancer cells themselves direct tissue remodelling, or whether this is performed exclusively through the recruitment of fibroblasts [12,13]. This stiffening of the tumour tissue and its stroma can act as a physical barrier, hindering the delivery of nutrients and oxygen, resulting in a hypoxic environment. Whilst this lack of oxygen can lead to tissue and tumour necrosis, it will simultaneously influence cellular adaptation and stimulate angiogenesis, promoting cancer cell survival. Often, this barrier can also act as a hindrance to immune infiltration and additionally impairs drug delivery, resulting in unprecedented resistance to therapy [7]. The diffusion of oxygen through a 3D tumour model from the surface to the core creates a hypoxic gradient mimicking that of an in vivo tumour (where oxygen diffuses from the high concentration near the vascular supply into the distant tumour hypoxic core [14]). As the rate of cellular oxygen consumption and the rate of oxygen diffusion reaches a steady state of equilibrium, a hypoxia gradient is generated since the rate of oxygen consumption is faster than the rate of oxygen diffusion [15].

One of the most researched pathways that permit invasion and metastasis is a process described as epithelial to mesenchymal transition (EMT). EMT is a process by which cancer cells undergo sequential alterations in which they gradually lose epithelial features and concurrently gain a mesenchymal phenotype and cellular markers [16]. A mesenchymal phenotype enables cancer cells to invade and metastasize into distant organs. Once a metastatic cell reaches a favourable environment, it reverses the cascade to undergo mesenchymal to epithelial transition (MET) and assumes an epithelial phenotype resembling the tissue of origin [17]. This newly derived idea of plasticity in cancer cells has given rise to the idea of cancer stem cells (CSCs), which are believed to be clonal subpopulations arising within tumours that can switch between phenotypes according to their environmental parameters [18]. A recent paper outlines a clear consensus statement on what constitutes EMT [19]. This states that EMT encompasses dynamic changes in cellular organization from down-regulation of epithelial features and concurrent acquisition of mesenchymal phenotypes resulting in functional changes to cell migration and invasion.

**Figure 1:**
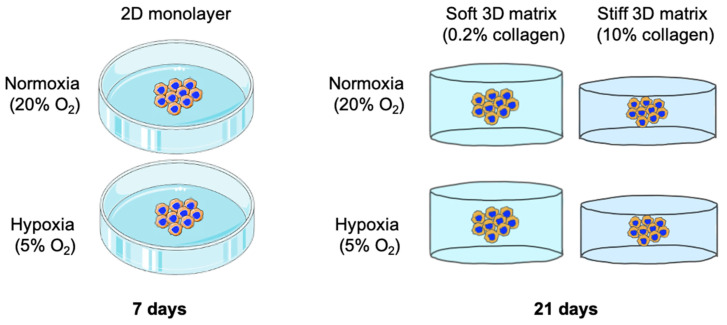
Experimental set up for HT-29 and MDA-MB-231 cells grown in 2D and 3D. For the 2D set up, cells were grown in either 21% or 5% O_2_ for 7 days and analysed for morphology, invasion and gene expression. For the 3D set up, cells were grown in either 21% or 5% O_2_ and in either a soft (0.2% collagen) or stiff (10% collagen) matrix for 21 days. After this, the samples were subsequently analysed for morphology and gene expression. Adapted using SMART-Servier Medical ART.

This study investigates whether two specific biophysical stimuli can promote an invasive phenotype or trigger the upregulation of EMT markers in the epithelial cancer cells HT-29 [20]. Namely, the comparison was made between commonly used collagen hydrogels (0.2% collagen) and plastic-compressed collagen [21,22] (10% collagen). Furthermore, the influence of physiological hypoxia on EMT status was considered. The breast cancer cell line MDA-MB-231 [23] was used as a control cell line as it is known to display EMT gene markers and present a strong mesenchymal-like phenotype [24,25,26]. This is shown in Figure 1.

The impact of the physical microenvironment on both cancer invasion and EMT will aid the development and optimisation of biomimetic and relevant, tissue-engineered, cancer–stroma models.

## 2. Results

### 2.1. Plastic Compression of Collagen Significantly Increased Stiffness as Measured through Atomic Force Microscopy (AFM)

**Figure 2:**
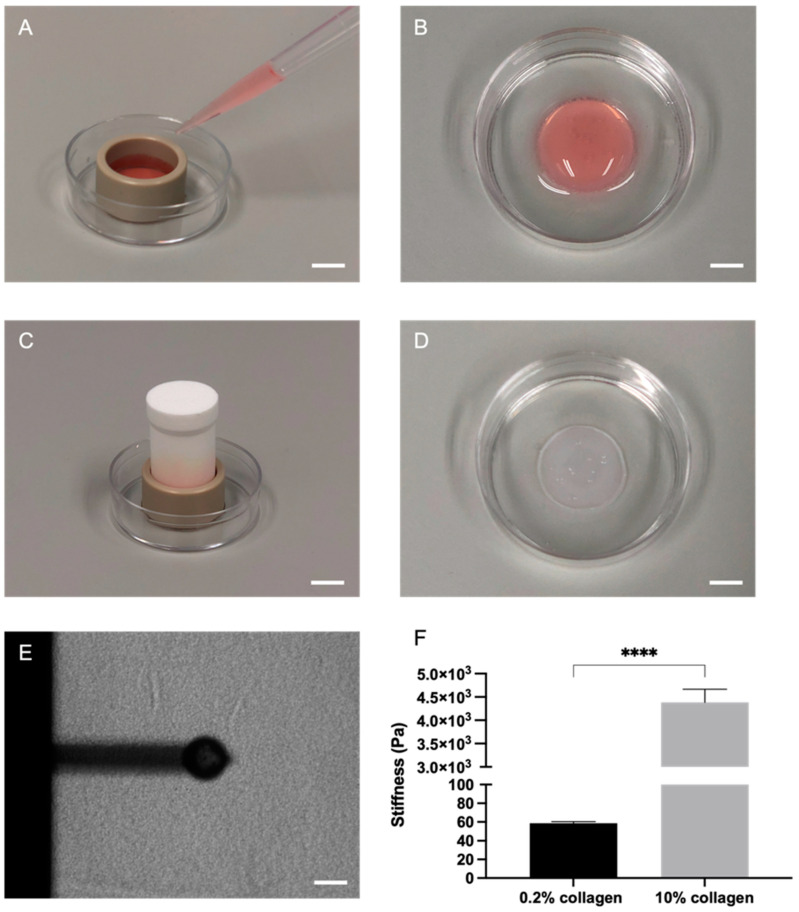
Atomic force microscopy (AFM) of collagen gels with varying collagen concentrations. (**A**) Casting of collagen gels within a polyetheretherketone (PEEK) ring. (**B**) Soft hydrogel (0.2% collagen) within petri dish. (**C**) Plastic compression with RAFT^TM^ absorbers. (**D**) Stiff plastic-compressed gel (10% collagen) within petri dish. Scale bar = 10 mm for (**A**,**C**) and 5 mm for (**B**,**D**). (**E**) Positioning of cantilever with glass bead. Scale bar = 50 µm. (**F**) Stiffness (Young’s modulus (**E**), Pa) of acellular 0.2% collagen and 10% collagen gels as measured through AFM. Mann-Whitney significance is shown. All *p*-value significance is indicated as: **** *p* < 0.00005.

Stiffness measurements were conducted using AFM on the soft 0.2% collagen hydrogels and the stiff 10% collagen gels. The collagen was cast in polyetheretherketone (PEEK) moulds for ease (Figure 2A–D) and measured by a 2 N/m cantilever with a glass bead attached (Figure 2E). As demonstrated in Figure 2F, the stiffness of the soft 0.2% hydrogels was 58.7 ± 5.64 Pa, and for the 10% collagen gels the stiffness was 4.38 ± 1.45 kPa. This difference was statistically significant (*p* < 0.0001).

### 2.2. The Inherent EMT Status of HT-29 Cells and MDA-MB-231 Epithelial Cancer Cells

HT-29 cells are epithelial cancer cells that have yet to undergo EMT. The epithelial cancer cells MDA-MB-231, however, inherently present strong mesenchymal-like features, suggesting they have already undergone EMT at the time of the cell harvest and immortalisation, hence serving as a positive control for EMT. This is demonstrated herein, as well as extensively in the literature [24,25,26].

**Figure 3:**
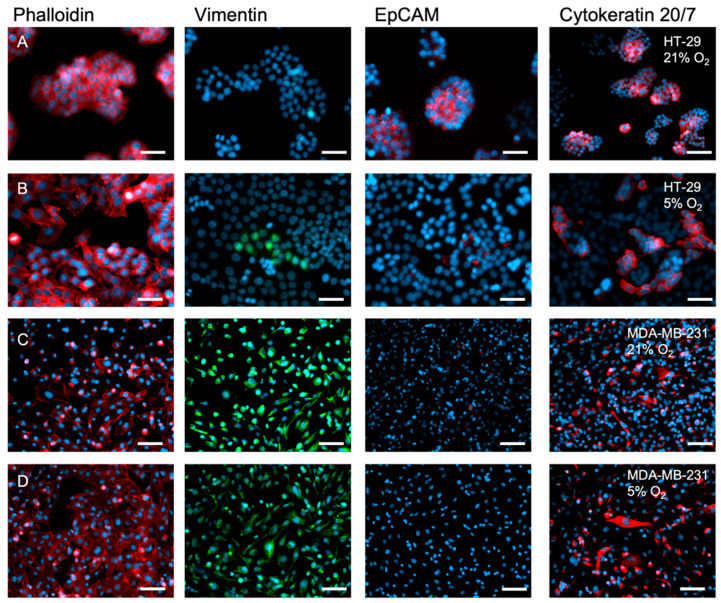
EMT markers in 2D monolayers of HT-29 and MDA-MB-231 cells cultured within differing oxygen environments. (**A**) HT-29 cells grown for 7 days in normoxia (21% O_2_). (**B**) HT-29 grown for 7 days physiological hypoxia (5% O_2_). (**C**) MDA-MB-231 cells grown for 7 days in normoxia (21% O_2_). (**D**) MDA-MB-231 cells in physiological hypoxia (5% O_2_). Red = Phalloidin, EpCAM, or CK20/7; green = Vimentin; blue = DAPI; and scale bar = 50 µm.

**Figure 4:**
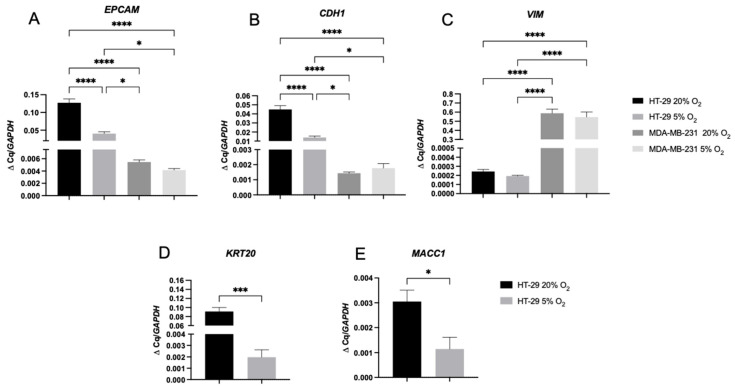
EMT gene marker expression within HT-29 and MDA-MB-231 2D monolayers cultured for 7 days in either normoxia (21% O_2_) or physiological hypoxia (5% O_2_). mRNA expression of (**A**) Epithelial cell adhesion marker (*EPCAM*), (**B**) E-cadherin (*CDH1*), (**C**) Vimentin (*VIM*), (**D**) Cytokeratin 20 (*KRT20*), and (**E**) Metastasis-associated in colon cancer protein 1 (*MACC1*). The fold change shown is normalized relative to reference gene glyceraldehyde-3-phosphate dehydrogenase (*GAPDH*). One-way ANOVA significance with Dunnet’s post hoc correction is shown for *VIM, EPCAM*, and *CDH1* with DOF = 11 for all and F-value = 79.5, 95.3, and 82.6, respectively. Unpaired *t*-test significance is shown for *KRT20* and *MACC1* with DOF = 4 for both and t-value= 4.413 and 2.917, respectively. All *p*-value significance is indicated as: * *p* < 0.05, *** *p* < 0.0005, **** *p* < 0.00005.

Figure 3A (2D, 21% O_2_) shows, via staining, that HT-29 cells strongly expressed epithelial cell adhesion marker EpCAM. Vimentin, a mesenchymal marker, is not expressed by HT-29 cells. MDA-MB-231 cells, however, expressed low EpCAM and high vimentin (Figure 3C). Gene expression of EpCAM (*EPCAM*), E-cadherin (*CDH1*), and vimentin (*VIM*) when the cells were cultured in 2D, 21% O_2_ support the IHC staining (Figure 4A–C). *EPCAM* expression was significantly higher in HT-29 cells (100-fold higher, *p* < 0.0001), and so was *CDH1* (10-fold, *p* < 0.0001). Both are epithelial markers. VIM expression, however, was 1000-fold higher in MDA-MB-231 cells (*p* < 0.0001).

### 2.3. A 2D Hypoxic Environment Triggered EMT in HT-29 Evidenced by Decreased EPCAM and KRT20 Expression

HT-29 cells started to display a mesenchymal phenotype after being cultured in 5% O_2_ for 7 days in 2D monolayers. The cell’s phenotype started to appear elongated and spindle-like when stained for Phalloidin/DAPI, leaning towards a more mesenchymal morphology compared to a more rounded, epithelial phenotype under 21% O_2_ (Figure 3A,B). Furthermore, the HT-29 cells lost epithelial cell adhesion marker EpCAM, as shown by the EpCAM antibody fluorescence levels, but also by the lack of cell clusters in 5% O_2_ due to the loss of the cell–cell adhesion capacity. Cytokeratin 20 expression was also lost, whilst vimentin expression appeared in a limited number of clonal subpopulations by day 7. The control cell type, MDA-MB-231 cells, showed no change in phenotypic markers when cultured in different oxygen concentrations and displayed a mesenchymal phenotype when grown under 5% or 21% O_2_. Gene markers associated with these two phenotypes were indicative of the presence or absence of EMT. As seen in Figure 4C, vimentin (*VIM*) expression was low within the HT-29 cells under both oxygen concentrations, compared to the MDA-MB-231 cells with established EMT status [24,25,26]. *EPCAM* expression (Figure 4A) in HT-29 cells was high; however, this expression decreased in the HT-29 cells when cultured in 5% O_2_ (*p* < 0.0001).

*CDH1* (Figure 4B) was also statistically significantly downregulated for HT-29 cells grown in 5% O_2_ (*p* < 0.0001). Both of these relate to the observed loss of EpCAM staining in HT-29 cells cultured in 5% O_2_ [27]. Keratin (*KRT20*) expression was downregulated (Figure 4D) to a statically significant level when cultured in 5% O_2_ (*p* = 0.0005 for *KRT20*). The metastasis-associated in colon cancer protein 1 *MACC1* (Figure 4E) was also upregulated in the HT-29 cells grown in 21% O_2_ to a statistically significant level (*p* = 0.0434).

### 2.4. A 3D Stiff Environment at Physiological Hypoxia Induced an Invasive Phenotype and Genotype in HT-29 Cells

**Figure 5:**
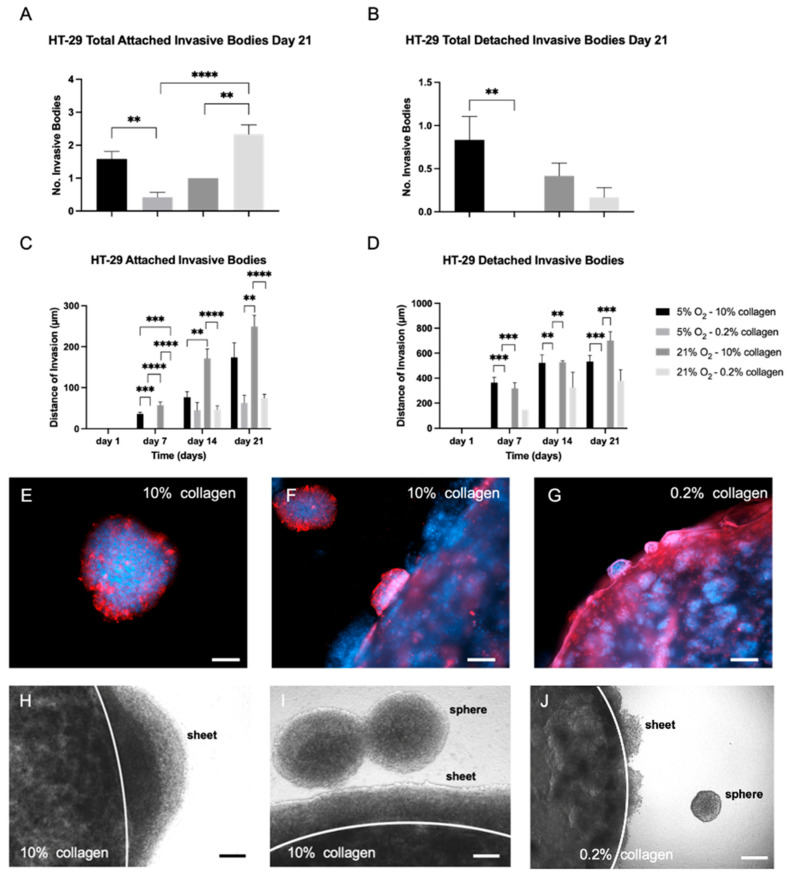
Number of invasive bodies and distance of invasion in HT-29 tumouroids with varying stiffness and oxygen concentrations. HT-29 tumouroids showed attached and detached invasive bodies. (**A**) Total number of attached invasive bodies within HT-29 tumouroids by day 21. (**B**) Total number of detached invasive bodies within HT-29 tumouroids by day 21. (**C**) Distance of invasion into the stromal compartment by attached invasive bodies over time within HT-29 tumouroids. (**D**) Distance of invasion into the stromal compartment by detached invasive bodies over time within HT-29 tumouroids. (**E**,**F**) Examples of invasive bodies into the stromal compartment within stiff 10% collagen tumouroids. (**G**) Example of minimal invasion into the stromal compartment of soft 0.2% collagen. Red = CK20; blue = DAPI; and scale bar = 50 µm for (**E**); 100 µm for (**F**); and 500 µm for (**G**). (**H**,**I**) Brightfield images of invasive bodies into the stromal compartment of stiff 10% collagen and (**J**) 0.2% collagen tumouroids. Scale bar = 100 µm for all. Significance shown for Kruskal–Wallis multiple comparisons test with Dunn’s post hoc correction. All *p*-value significance is indicated as: ** *p* < 0.005, *** *p* < 0.0005, **** *p* < 0.00005.

**Figure 6:**
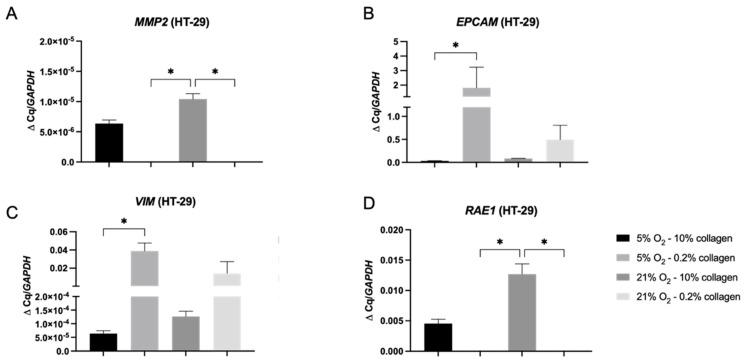
Gene expression within HT-29 tumouroids grown in differing collagen and oxygen concentrations. (**A**) *MMP2*, (**B**) *EPCAM*, (**C**) *VIM*, and (**D**) *RAE1* mRNA levels expressed by day 21 in HT-29 tumouroids within stiff (10% collagen) or soft (0.2% collagen) and cultured under physiological hypoxia (5% O_2_) or normoxia (21% O_2_). The fold change shown is normalized relative to reference gene *GAPDH*. Significance shown for Kruskal–Wallis multiple comparisons test with Dunn’s post hoc correction. All *p*-value significance is indicated as: * *p* < 0.05.

The number of invasive bodies formed by HT-29 after 21 days in a 3D collagen matrix (soft or stiff) and their distance invaded into the acellular stroma can be seen in Figure 5. Multiple parameters were assessed to provide an in-depth understanding of invasion, including type (attached or detached), distance, and surface area. HT-29 cells formed attached invasive bodies in all conditions; however, detached bodies were almost exclusively observed in stiff collagen. The number of attached invasive bodies can be seen in Figure 5A. The highest number of detached invasive bodies was observed in physiological hypoxia in the stiff stroma (5% O_2_ and 10% collagen), as seen in Figure 5B. This correlates with the *MMP2* gene data in Figure 6, which shows *MMP2*, an invasion marker, is upregulated in HT-29 cells in the stiff (10% collagen) gels.

The distance of invasion was greatest for both the attached and detached invasive bodies when cultured in normoxia in the stiff stroma (21% O_2_ and 10% collagen) (Figure 5C,D). This is also confirmed with high *MMP2* gene upregulation in stiff collagen cultures at 21% O_2_. Phenotypically, this can be observed in Figure 5E–G which show examples of invasive bodies. In terms of surface area of invasion, the HT-29 cells invaded the widest in normoxia within the stiff stroma (21% O_2_ and 10% collagen). This was the case for the attached and detached invasive bodies (Supplementary Figure S1). The second highest area of invasion was within physiological hypoxia within stiff stroma (5% O_2_ and 10% collagen). The HT-29 cells invaded as sheets and detached spheres in both the soft and stiff collagen stroma (Figure 5H–J). All quantified values and descriptive statistics for the invasive measures can be taken from Supplementary Tables S1 and S2.

The *MMP2* gene (Figure 6A) was statistically significantly upregulated in the stiff 10% collagen matrix when compared to the soft 0.2% collagen matrix (*p* = 0.039) for both oxygen conditions. In terms of EMT gene markers, *EPCAM* and *VIM* (Figure 6B,C) were statistically significantly upregulated in physiological hypoxia within a soft matrix (5% O_2_ and 0.2% collagen) (*p* = 0.0279 for both) indicating a mixed cell population. However there is a clear link that cells in the stiff 10% collagen, which invade the greatest using all parameters, display high gene expression of *MMP2* and vimentin, whilst low expression of *EPCAM* (Figure 6). Ribonucleic acid export 1 *RAE1* (Figure 6D) was statistically significantly upregulated in the stiff 10% collagen matrix when compared to the soft 0.2% collagen matrix (*p* = 0.0392) in normoxia (21% O_2_), indicating an aggressive EMT genotype of the cells.

### 2.5. EMT Was Not Reversed by Biophysical Stimuli, Evidenced by MDA-MB-231 Cells, Which Showed Insignificant Changes in Invasion in Different 3D Microenvironments

**Figure 7:**
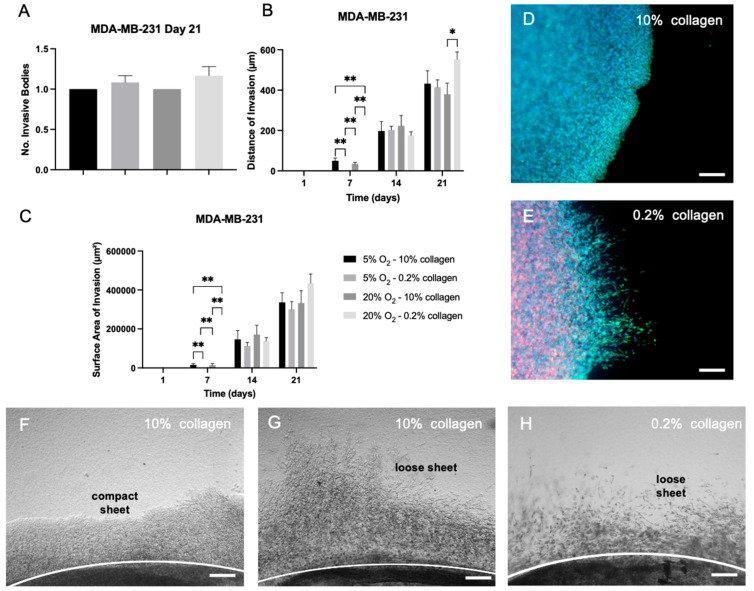
Quantitative measurements of invasion for MDA-MB-231 cells in 3D with varying stiffness and under differing oxygen concentrations. MDA-MB-231 tumouroids showed attached invasive bodies only within selected regions of interest near the artificial cancer mass (ACM). (**A**) Total number of attached invasive bodies within MDA-MB-231 tumouroids by day 21. (**B**) Distance of invasion into the stromal compartment by attached invasive bodies over time within MDA-MB-231 tumouroids. (**C**) Surface area of invasion of attached invasive bodies within MDA-MB-231 tumouroids. (**D**) Invasion of cells into stiff 10% collagen tumouroids in a compact sheet formation. (**E**) Invasion of cells into a soft 0.2% collagen stromal compartment showing a loose phenotype with low cell-to-cell attachment. (**F**,**G**) Brightfield images of cells invading as both compact and loose sheets into stiff 10% collagen tumouroids. (**H**) Cells invaded exclusively as very loose sheets into the soft 0.2% collagen stromal compartment. Red = CK7; green = Vimentin; blue = DAPI; and scale bar = 100 µm for all. Significance shown for Kruskal–Wallis multiple comparisons test with Dunn’s post hoc correction. All *p*-value significance is indicated as: * *p* < 0.05, and ** *p* < 0.005.

The MDA-MB-231 cells invaded solely as attached invasive bodies. There was no significant difference in the number of invasive bodies for the different physiological environments by day 21 (Figure 7A). In terms of distance of invasion (Figure 7B), there was little difference between conditions, with some statistically significant difference between this group and the cells grown in normoxia within a stiff matrix (21% O_2_ and10% collagen) (*p* = 0.0404) by day 21. For the surface area of invasion, there was no significant difference between the groups (Figure 7C) by day 21. The attached invasive sheets existed as compact and loose sheets (Figure 7F,G). They were observed to form both compact and loose sheets in the stiff 10% collagen (Figure 7F–H), whilst solely forming loose invasive sheets in the soft 0.2% collagen (Figure 7H). All descriptive statistics for the invasive measures can be taken from Supplementary Table S3.

## 3. Discussion

The main aim of this study was to investigate whether oxygen concentration and matrix stiffness of 3D tumour models would affect expression of some defined EMT markers and the consequential cancer invasion within an epithelial cancer cell (HT-29), compared to a cell line (MDA-MB-231) with established and well-recognised mesenchymal status [24,25,26]. Cells were initially grown in 2D in 5% or 21% O_2_. Then, these two cell lines were cultured in 3D, by incorporation into either a soft 0.2% collagen or stiff 10% collagen matrix, in either hypoxia or normoxia. Invasion patterns and gene expression were then quantified and analysed. The main conclusion is that a low oxygen concentration (physiological hypoxia) and a high matrix stiffness induced a more invasive, mesenchymal-like phenotype in HT-29 cells, whilst MDA-MB-231 cells were not impacted by physical parameters. Furthermore, the invasive marker, *MMP2*, was highly expressed when HT-29 cells were cultured in 10% collagen matrices, regardless of hypoxic status. This invasive marker was not expressed in the soft, 0.2% collagen matrix and therefore in this model, stiffness as a biophysical feature is a more powerful stimuli of invasion compared to hypoxia in the HT-29 cells.

Epithelial to mesenchymal transition (EMT) is a reversible process whereby cancer epithelial cells undergo defined phenotypic and genotypic changes to acquire mesenchymal properties to make them motile. The main markers used to assess EMT status are epithelial markers, including cytokeratins, E-cadherin, and EpCAM and mesenchymal markers, primarily vimentin. Markers of invasion in cancer are multiple, but the matrix metallo-proteases (MMP2) are key in this process. However, it must be noted that there exists great diversity of EMT phenotypic manifestations and, therefore, for any one single study to assess all features is difficult.

It was demonstrated that when the two cancer cell types were cultured in either oxygen concentration for 7 days in 2D, markers of EMT were upregulated in the HT-29 cells under physiological hypoxia at 5% O_2_. This was clearly demonstrated in the staining shown in Figure 3. HT-29 cells started to have a more elongated, mesenchymal phenotype. HT-29 cells lost CK20 and EpCAM expression but gained vimentin expression in some cell clusters. The gene data validate this, as *CDH1* is the gene for E-cadherin, and this was significantly downregulated in HT-29 cells when these cells were cultured in 5% O_2_. The gene expression in Figure 4B,C also showed the *EPCAM* and *KRT20* genes were significantly downregulated. Therefore in a 2D setting, hypoxia is clearly able to stimulate this change in gene expression. In comparison, the MDA-MB-231 cells genotype and phenotype are not influenced by these biophysical parameters.

The conclusions to be drawn from the HT-29 cells 3D data for invasion are complex and dependent on the micro-environmental biophysical parameters. For the MDA-MB-231 control cells, the biophysical parameters confer no difference to the invasion pattern or rate. Within the colorectal tumouroids, it was clear from Figure 5 that a stiff matrix (10% collagen) enhanced invasion. The stiff collagen 10% induced the greatest invasion measured (type, distance, and surface area) in HT-29 cells, regardless of oxygen. This was confirmed with high gene expression of *MMP2*, high expression of *VIM* gene, and low expression of *EPCAM*. Other groups working on 3D colorectal tumour models have also stressed the importance of modelling a biomimetic stiffness, especially in the initial stages of tumour development [28]. The gene data in Figure 6 indicates that HT-29 cells adopted a more aggressive genotype (high *MMP2*, high *VIM*, and low *EPCAM*) in the stiff collagen matrix. The *RAE1* gene specifically has been shown to induce aggressive cancer phenotypes [29] by mediating EMT [30] via *ZEB1* expression [31]. It is most likely the case that within the tumouroids, epithelial and mesenchymal cellular populations may co-exist [32], which could explain why EpCAM and vimentin were upregulated in the same conditions. Future explorative studies could therefore either sort the cells via flow cytometry to obtain a clearer idea of the phenotype and look at single-cell RNA sequencing in order to zoom in on the genotype distribution of the cells.

For the MDA-MB-231 cells in the 3D matrix, the invasion patterns observed in Figure 7 did not differ significantly within the different oxygen and collagen concentrations. This leads to the conclusion that these cells are most likely able to invade into a range of collagen densities and nutrient deprived regions, further underlining their aggressive behaviour [33]. Triple negative breast cancers, especially, pose one of the greatest challenges in biomedical research and modern medicine. Due to their increased propensity to drug resistance, it is therefore vital to model the disease appropriately [34]. Three-dimensional models rarely try and recreate the changing environments these cells can grow and metastasise into.

The invasive patterns of cancers have been of interest for some time. Patterns descried in literature tend to be either collective migration (sheet-like) or single cell, amoeboid-like migration [35]. Although both invasion types have been associated with tumour growth and metastatic potential, the collective sheet migration relies upon heterogeneity in the cells, where “leader” cells display specific receptors, including CXCR4 and CXCR7 [35]. The attached morphology described here is akin to the collective migration, compared to detached bodies. A recent paper describing tumour invasion patterns with prognosis depicts a broad pushing pattern as the least invasive, invasion of strands and cords as mid invasive, and invasive islands indicative of worst prognosis [35].

The tumouroid model provides a 3D system where the invasion patterns of outgrowing cancer or tumour cells can be visualised and analysed. This is in part due to the low water content of compressed collagen. As such, the patterns of attached and detached bodies have been described in this model previously [8]. We hypothesise that, as attached bodies are described in all set-ups, it is the detached body formation which indicates a more invasive phenotype. Detached body formation was only observed in the 10% collagen gels and corresponded with increased *MMP2* and *RAE1* gene expression. This model therefore adds to the growing literature around how we define EMT.

In terms of future directions, it is important to put the data into clinical perspective. Colon carcinoma tissue has a stiffness of around 1–10 kPa [36]. The 10% collagen matrix, at a stiffness of ~4 kPa, fulfilled this requirement, which was validated by the data as the cells preferentially grew into this matrix. As such, a more biomimetic stiffness should be used in 3D models of adenocarcinoma. It is therefore essential for future studies to test a range of stiffnesses and oxygen gradients when studying cancer cell behaviour.

## 4. Materials and Methods

### 4.1. Atomic Force Microscopy (AFM)

Acellular collagen type I gels were cast in a 3D-printed PEEK (polyetheretherketone) ring the size of a 24-well plate (custom made by PBH Engineering Ltd., Hertfordshire, UK) and placed in a 35 mm petri dish. This casting method was optimised specifically for AFM. To obtain accurate AFM measurements, the samples must be completely attached to the dish or a glass slide, as transferring the gel to a petri dish the day of imaging is not ideal. The collagen mix within the rings was left to cross-link at 37 °C for 15 min (min). Subsequently, half of the gels were left as 0.2% hydrogels and the other half were plastic compressed for 15 min using the 24-well RAFT^TM^ absorbers (Lonza, Slough, UK) to produce high-density 10% collagen gels. The 3D-printed rings were then delicately removed. The gels were stored in 2 mL of DMEM (Gibco^TM^ through Fisher Scientific, Loughborough, UK) at 5% CO_2_ atmospheric pressure and 37 °C until measured. To measure stiffness, a CellHesion^®^ 200 AFM (JPK BioAFM, Bruker Nano GmbH, Berlin, Germany) was used. Measurements were performed at room temperature, in Leibovitz’s L-15 Medium, no phenol red (Gibco^TM^ through Thermo Fisher Scientific, Loughborough, UK). A stiff cantilever with a spring constant of approximately 2 N/m (RFESP-75, Bruker, Berlin, Germany) with a 50 µm glued glass bead (Cospheric LLC, Goleta, CA, USA) was used to probe the samples. Each sample was probed along a 4 × 4 map of 2000 × 2000 µm, leading to a total of 16 measurements per sample. The set force was 50 nN on the 0.2% hydrogels and 700 nN on the 10% compressed gels to ensure a 10 to 15 µm indentation. Using the JPK BioAFM SPM data processing software, the Hertz model was fitted to the collected force curves to determine the Young’s Modulus E, assuming a Poisson ratio (ν) of 0.5.

### 4.2. Cancer Cell Culture

HT-29 and MDA-MB-231 immortalised cell lines were both acquired through the European Collection of Authenticated Cell Cultures (Sigma-Aldrich, Dorset, UK). The cells were routinely cultured in Dulbecco’s Modified Eagle Medium (DMEM) at 1000 mg/L glucose. The DMEM was supplemented with 10% foetal bovine serum (FBS) and 100 units/mL of penicillin and 100 µg/mL streptomycin. The cells were passaged twice a week at a 1/4–1/10 dilution with the dissociating agent TrypLE^TM^ following two phosphate buffered saline (PBS) washes (all Gibco^TM^ through Thermo Fisher Scientific, Loughborough, UK). For normoxia conditions, the cells were cultivated in 5% CO_2_, resulting in a ~21% atmospheric O_2_ environment. For the physiological hypoxia, the cells were cultivated in a hypoxia incubator (Sanyo multi-gas incubator, MCO-18M). Here, the settings are set to 5% O_2_ and are achieved by using nitrogen to purge excess O_2_ from the incubator. This is checked by an incubator sensor, which allows for additional nitrogen gas until the 5% O_2_ was reached. The limitation to our study was that due to the lack of a hypoxia workstation, every 72 h media changes were conducted in ambient O_2_ conditions. The 2D samples were cultured in either O_2_ condition for 7 days with no media change at a 2 × 10^5^ cellular density per 6-well. All tumouroids were cultured for 21 days in either oxygen condition with 50% media changes every 48–72 h (h). All cells were grown at 37 °C.

### 4.3. D Tumouroid Set-Up

In order to create “tumouroids” as previously described [37], the HT-29 and MDA-MB-231 cells were firstly incorporated into an artificial cancer mass (ACM). Briefly, monomeric collagen type I of rat tail origin (First Link, Birmingham, UK) is mixed with 10× MEM (Gibco^TM^ through Thermo Fisher Scientific, Loughborough, UK) and neutralising agent (N.A.) according to the RAFT^TM^ protocol. The desired cellular concentration, in this case 5 × 10^4^/ACM, is added into a set volume of DMEM to the collagen/10× MEM/N.A. mix. In order to cross-link the collagen, 240 µL of the cellular collagen mix is suspended into a 96-well plate and incubated at 37 °C for 15 min. Plastic compression is then utilised to withdraw the water content from these hydrogels using the 96-well size RAFT^TM^ absorbers (Lonza, Slough, UK) for 15 min. This results in the collagen concentration increasing from 0.2% to ~10% [8]. This ACM was then implanted into a stromal compartment of either a soft (0.2%) or stiff (10%) collagen density. For both, 50% of the total 1.3 mL acellular stromal collagen mix was suspended into a 24-well plate and left to cross-link at 37 °C for 15 min. The ACM was then placed on top with fine tip tweezers (Fisherbrand^TM^ Dumont #5 through Thermo Fisher Scientific, Loughborough, UK) and the other 50% of the acellular stromal collagen mix w added and again left to cross-link at 37 °C for 15 min. In the case of the soft density stromal compartment (0.2% collagen), the tumouroids were left like this. In order to generate the stiff stromal compartment (10% collagen), the tumouroids were plastic compressed again as a whole for 15 min with the 24-well size RAFT^TM^ absorbers (Lonza, Slough, UK). This set-up can be seen in Figure 1.

### 4.4. Immunostaining

Two-dimensional monolayers were formalin fixed for 15 min, and 3D tumouroids were formalin fixed for 1 h with 10% neutrally buffered formalin. The samples were then washed twice with PBS and stored in PBS also. The constructs were permeabilised using PBS containing 0.2% Triton X-100 and 1% bovine serum albumin (BSA) for one hour at room temperature (both Sigma-Aldrich, Dorset, UK). Primary and secondary antibodies specifications and dilutions can be taken from Supplementary Table S4 below. In short, 1° antibody incubation was performed overnight at 4 °C. After three 5 min PBS washes on the plate shaker, the 2° antibody was then incubated for 2.5 h at room temperature. After this, the samples were washed three times for 10 min on the plate shaker after five quick washes. For phalloidin staining, the samples were incubated for 30 min and washed once with PBS. Finally, the samples were counterstained with 4′,6-diamidino-2-phenylindole (DAPI) to visualise the nucleus.

### 4.5. Measurements of the Invasive Phenotype

All fluorescent images for phenotype and measurements were taken on the Zeiss AxioObserver using the Zeiss ZEN software (version 3.4, Zeiss, Oberkochen, Germany). Four images within the regions of interest (ROI) were taken as previously described [37]. Within these images, the number of detached and attached invasive bodies was quantified additionally to their distance of invasion (µm) from the original ACM and their surface area (µm^2^). These measurements were conducted in the Fiji ImageJ software (version 2.1.0) [38].

### 4.6. RNA Isolation and Quantitative Polymerase Chain Reaction (qPCR)

After initial lysis of the samples in TRI Reagent^TM^, RNA was extracted using the TRIzol-Chloroform phase separation method [39] (both Sigma-Aldrich, Dorset, UK). The extracted RNA was quantified and checked for purity using the NanoDrop^TM^. The RNA was transcribed into complementary DNA (cDNA) using the High-Capacity cDNA Reverse Transcription Kit (Applied Biosystems^TM^ through Thermo Fisher Scientific, Loughborough, UK) on the T100^TM^ Thermal Cycler (Bio-Rad, Watford, UK). Primers were designed in line with the MIQE guidelines [40]. All primer pairs fulfilled the following parameters: exon–exon spanning (see Supplementary Figure S2), an annealing temperature (Ta) of 60 °C, a primer sequence length of 20–25 base pairs (bp), a product length of 100–200 bp, a maximum self-complementarity of 5 and maximum 3′ self-complementarity of 3. Additionally, the primer pairs had a maximum poly-x value of 3 or less and one GC clamp. Primer pairs were checked for secondary structure under SYBR^TM^ ionic conditions at 60 °C using mFold. Finally, the primer pairs were run through Primer-BLAST to check for specificity. The newly designed primer pair sequences and efficiencies can be found in Supplementary Table S5, below, additionally to the primer pairs taken from literature. All primers were ordered through Eurofins Genomics (Ebersberg, Germany) and used at concentration of 0.2 µM. Genes of interest were amplified on the CFX96^TM^ Touch System utilising the iTaq^TM^ Universal SYBR^TM^ Green Supermix (both Bio-Rad, Watford, UK). Gene expression was calculated using the ΔCt method [41]. All genes of interest were normalised to glyceraldehyde-3-phosphate dehydrogenase (GAPDH) expression.

### 4.7. Statistical Analysis

All data was collected on a minimum of n = 3 biological replicates. qPCR data included a n = 3 of technical replicates on top of the n = 3 biological replicates. All measures of invasion was calculated based on four randomised ROIs per biological replicate. All data was analysed and visualised using GraphPad Prism 9 software. First, all data sets were tested for normal distribution using the Shapiro–Wilk test (n = 3–7) or the D’Agostino test (n ≥ 8). The appropriate statistical test was then decided upon. In general, for parametric data sets, the unpaired *t* test and one-way ANOVA with Dunnet’s post hoc correction were used to compare between two groups or for multiple comparisons respectively. For non-parametric data sets, the Mann–Whitney or Kruskal–Wallis test with Dunn’s multiple comparison correction were used in the same manner. The statistical tests used are stated in each figure legend, additionally to F values, t-values, and degrees of freedom (DOF). Statistical significance was accepted at *p* value <0.05 and all data points are represented at mean with standard error mean (SEM) in graphs as stated in text and tables as mean with standard deviation (STDEV).

## Data Availability

All data is available upon reasonable request from U.C.

## Supplementary Materials

The supporting information can be downloaded at: https://www.mdpi.com/article/10.3390/ijms24043956/s1.
