# Supplementary material for: Biophysical Parameters Can Induce Epithelial-to-Mesenchymal Phenotypic and Genotypic Changes in HT-29 Cells: A Preliminary Study
Source: Int J Mol Sci. 2023 Feb 16;24(4):3956. doi: 10.3390/ijms24043956 (PMC9962772; doi:10.3390/ijms24043956)
Supplement: Supplementary file 1 [file ijms-24-03956-s001.zip › ijms-2161903-supplementary.pdf]

Supplementary Material

Biophysical Parameters Can Induce Epithelial-to-Mesenchymal Phenotypic and Genotypic Changes in HT-29 Cells: A Preliminary Study

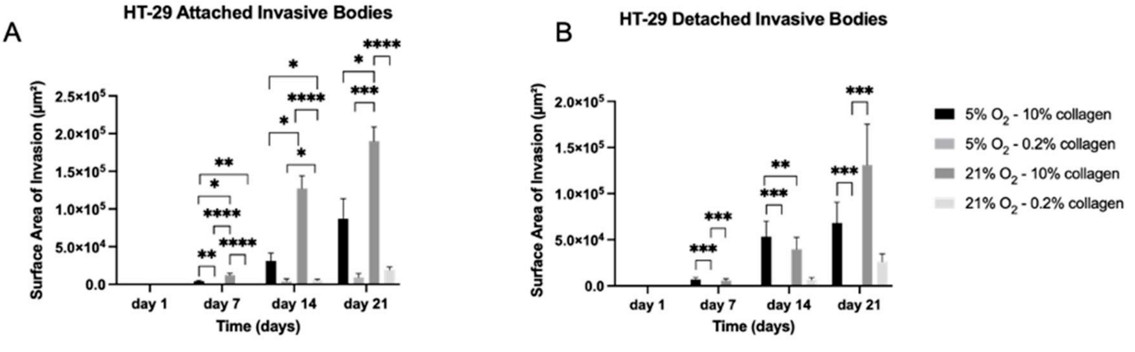

Supplementary Figure S1. Surface area of invasive bodies within HT-29 tumouroids grown in varying collagen and oxygen concentrations. (A) Surface area of invasion by attached invasive bodies within HT-29 tumouroids. (B) Surface area of invasion by detached invasive bodies within HT-29 tumouroids. Significance shown for Kruskal-Wallis multiple comparisons test with Dunn's post hoc correction. All p-value significance is indicated as: * $p < 0.05$, ** $p < 0.005$, *** $p < 0.0005$ and **** $p < 0.00005$.

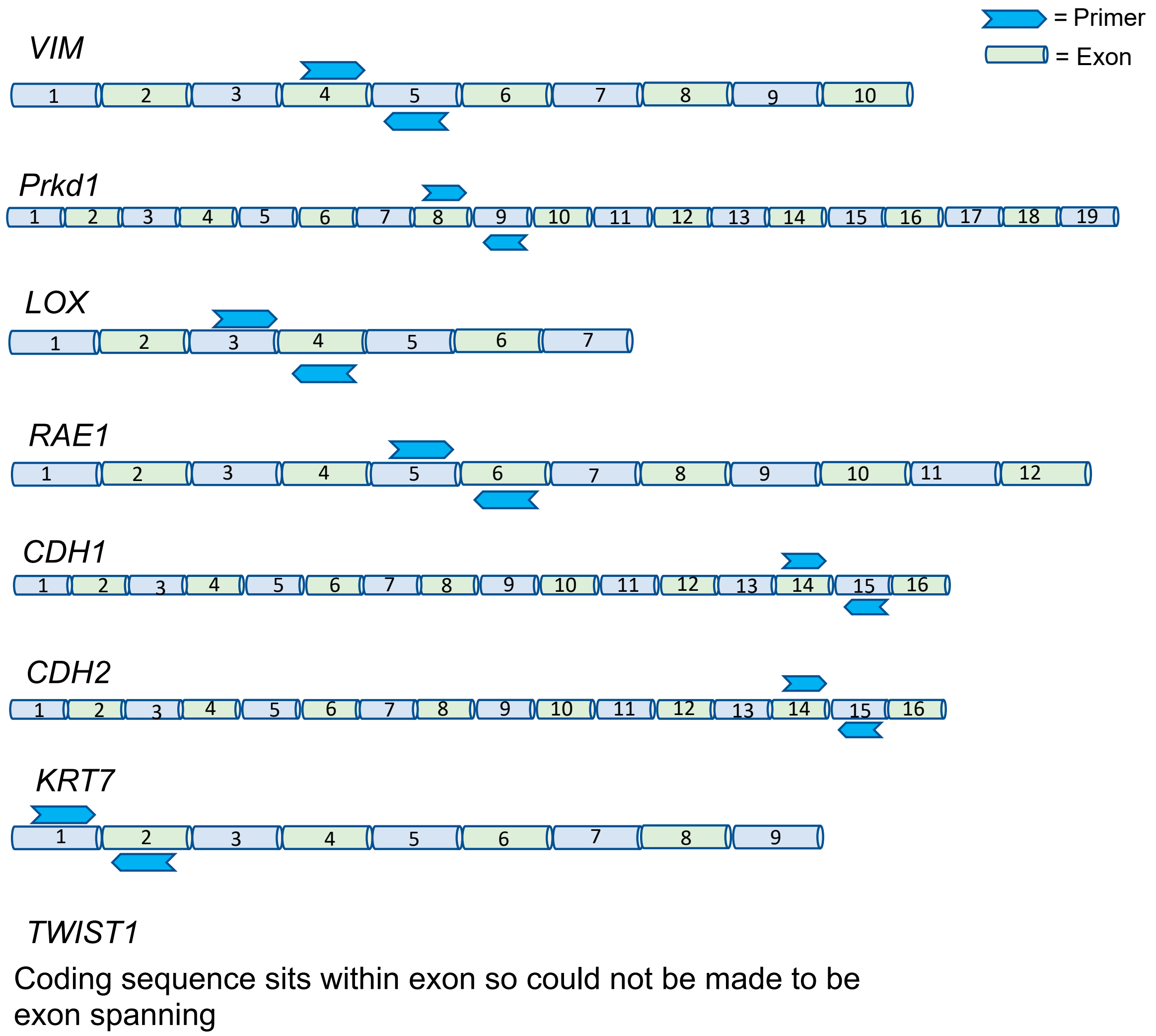

Supplementary Figure S2. Primer placement within exon-exon regions of the coding sequence for newly designed primer pairs.

Supplementary Table S1. Descriptive statistics for invasion measurements within HT-29 tumouroids. Attached invasive bodies only. Data is represented as mean ± STDEV.

**Day**	**7**	**14**	**21**
	**5% O_2 - 10% collagen**		
No. Invasive Bodies	1.92±1.31	2.25 ±1.14	1.58 ±0.793
Distance (μm)	35.8±20.0	76.7±68.0	174±155
Surface Area (μm²)	3.99x10³±6.69x10³	3.12x10⁴±5.27x10⁴	8.70x10⁴±11.7x10⁴
	**5% O_2 - 0.2% collagen**		
No. Invasive Bodies	0.00±0.00	0.167±0.389	0.417±0.515
Distance (μm)	0.00±0.00	44.8±26.7	62.5±42.0
Surface Area (μm²)	0.00±0.00	4.02x10³±4.52x10³	9.04x10⁴±1.21x10⁴
	**21% O_2 - 10% collagen**		
No. Invasive Bodies	2.17±1.40	1.08±0.289	1.0±0.0
Distance (μm)	57.1±39.5	171±84.5	249.6±93.54
Surface Area (μm²)	1.22x10⁴±1.37x10⁴	1.27x10⁵±6.04x10⁴	1.90x10⁵±6.49x10⁴
	**21% O_2 - 0.2% collagen**		
No. Invasive Bodies	0.00±0.00	1.58±1.24	2.33±0.9847

Distance (μm)	0.00±0.00	46.5±39.7	74.59±49.59
Surface Area (μm^2)	0.00±0.00	$5.58x10^{3}\pm5.61x10^{3}$	$1.92x10^{4}\pm1.98x10^{4}$

Supplementary Table S2. Descriptive statistics for invasion measurements within HT-29 tumouroids. Detached invasive bodies only. Data is represented as mean ± STDEV.

**Day**	**7**	**14**	**21**
	**5% O_2 - 10% collagen**		
No. Invasive Bodies	0.583± 0.996	0.500±0.522	0.833±0.937
Distance (μm)	365±113	522±157	533±157
Surface Area (μm^2)	$6.89x10^{3}\pm5.63x10^{3}$	$5.34x10^{4}\pm4.07x10^{4}$	$6.83x10^{4}\pm7.08x10^{4}$
	**5% O_2 - 0.2% collagen**		
No. Invasive Bodies	0.00±0.00	0.00±0.00	0.00±0.00
Distance (μm)	0.00±0.00	0.00±0.00	0.00±0.00
Surface Area (μm^2)	0.00±0.00	0.00±0.00	0.00±0.00
	**21% O_2 - 10% collagen**		
No. Invasive Bodies	0.750±1.29	0.417±0.669	0.417±0.515
Distance (μm)	318±138	528±24.8	701±158
Surface Area (μm^2)	$5.64x10^{3}\pm6.01x10^{3}$	$3.95x10^{4}\pm2.96x10^{4}$	$1.31x10^{5}\pm9.95x10^{4}$
	**21% O_2 - 0.2% collagen**		
No. Invasive Bodies	0.0833±0.289	0.167±0.389	0.167±0.389
Distance (μm)	148±0.00	325±173	379±126
Surface Area (μm^2)	486±0.00	$6.61x10^{3}\pm3.19x10^{3}$	$2.62x10^{4}\pm1.18x10^{4}$

Supplementary Table S3. Descriptive statistics for invasion measurements within MDA-MB-231 tumouroids. Attached invasive bodies only. Data is represented as mean ± STDEV.

**Day**	**7**	**14**	**21**
	**5% O_2 - 10% collagen**		
No. Invasive Bodies	0.750±0.622	1.17±0.389	1.00±0.00
Distance (μm)	50.4±47.8	198±174	432±220
Surface Area (μm^2)	$1.57x10^{4}\pm2.37x10^{4}$	$1.46x10^{5}\pm1.68x10^{5}$	$3.36x10^{5}\pm1.7x10^{5}$
**5% O_2 - 0.2% collagen**			
No. Invasive Bodies	0.00±0.00	1.17±0.389	1.08±0.289
Distance (μm)	0.00±0.00	203±67.8	414±129
Surface Area (μm^2)	0.00±0.00	$1.13x10^{5}\pm6.88x10^{4}$	$3.00x10^{5}\pm1.44x10^{5}$
	**21% O_2 - 10% collagen**		
No. Invasive Bodies	0.917±0.900	1.00±0.00	1.00±0.00
Distance (μm)	34.1±34.3	223±176	380±190
Surface Area (μm^2)	$1.45x10^{4}\pm3.40x10^{4}$	$1.71x10^{5}\pm1.66x10^{5}$	$3.33x10^{5}\pm2.20x10^{5}$
	**21% O_2 - 0.2% collagen**		
No. Invasive Bodies	0.00±0.00	1.17±0.390	1.17±0.390
Distance (μm)	0.00±0.00	176±68.0	553±137
Surface Area (μm^2)	0.00±0.00	$1.38x10^{5}\pm6.14x10^{4}$	$4.34x10^{5}\pm1.79x10^{5}$

Supplementary Table S4. Specifications and dilutions of 1° and 2° antibodies for immunofluorescent staining and counterstaining.

**Target**	**Details**	**Dilution**
**Cytokeratin 7**	Rabbit EPR17078 ab181598 (Abcam, Cambridge, UK)	All four at 1:200
**Cytokeratin 20**	Rabbit D9Z1Z (New England Biolabs, Hitchin UK)	
**EpCAM**	Rabbit EPR20532-225 ab223582 (Abcam, Cambridge, UK)	
**Vimentin**	Mouse V9 (Santa Cruz, Dallas, US)	

**Rabbit**	DyLight® 594 IgG H&L ab96885 (Abcam, Cambridge, UK)	
**Mouse**	Alexa Fluor™ 488 IgG H&L ab150113	Both at 1:1000
**Phalloidin**	Alexa Fluor™ 568 Phalloidin Kit (Invitrogen™ through Thermo Fisher Scientific, Loughborough, UK)	1.65 μM
**DAPI**	NucBlue™ (Invitrogen™ through Thermo Fisher Scientific, Loughborough, UK)	2 drops per mL

Supplementary Table S5. Primer sequences, amplicon size (bp) and efficiencies (%).

RAE1	TTTGCATACGCTTCCAGCTACG (22)	TTAGCTCTTCGGCTGCATTACG (22)	100	95.2
CDH1	GAAGAAGGAGGCGGAGAAGAGG (22)	CATGAGGGTTGGTGCAACGTC (21)	108	102
CDH2	GATAAAGAACGCCAGGCCAAAC (22)	CATCAGGCTCCACAGTGTCAGG (22)	151	97.0
KRT7	GGAGGAGAGCGAGCAGATCAAG (22)	AGCAATCTGGGCCTCAAAGATG (22)	172	90.2
KRT20	ATTGCTACTTACCGCCGCCTTC (22)	GACACGACCTTGCCATCCACTAC (23)	143	98.3
TWIST-1	GACTCCAAGATGGCAAGCTG (20)	CTAGTGGGACGCGGACATGG (20)	105	98.2
EpCAM [23]	TTGCTGTTATTGTGGTTGTGGTG (23)	CCCATCTCCTTTATCTCAGCCTTC (24)	112	101
MMP2 [9]	CAGGAGGAGAAGGCTGTGTTC (21)	TAAAGGCGGCATCCACTCG (19)	136	99.4
MACC1 [23]	TACGACTCACAAAGCAACAAATGG (24)	AAATCATAGGCAGGTTTCCACATC (24)	100	97.0
HIF-1α [9]	CCAGCAGACTCAAATACAAGAACC (24)	TGTGGGTAGGAGATGGAGATGC (22)	134	104
